# Adopting AI Enhances Humanitarian Operations While Demanding Critical Trade-Offs

**DOI:** 10.1055/s-0046-1822817

**Published:** 2026-06-26

**Authors:** Caroline Grangier, Munzer Alkhalil, Aula Abbara

**Affiliations:** 1 Antei Global, Paris, France; 2 Research for Health System Strengthening in Northern Syria (R4HSSS), The Centre for Conflict & Health Research (CCHR), King's College London, London, United Kingdom; 3 David Nott Foundation, London, United Kingdom; 4 Department of Infection, Imperial College London, London, United Kingdom; 5 Syria Public Health Network, London, United Kingdom

**Keywords:** AI, health, humanitarians, conflict zones, ethical considerations, trade-off

## Abstract

Artificial intelligence (AI) is increasingly impacting the cooperation sector, transforming how humanitarians are operating in the field. AI tools now allow for improved health diagnostics and quality services. In conflict areas, organizations are able to improve the efficiency of their analysis, and propose optimized data management processes for more multifactorial predictions. In a context where human and financial resources are limited, AI address the gaps to sustain emergency responses. In this commentary, we explore the trade-offs of adopting those tools and the ethical concerns their usages raise in a fragile environment. Security breach due to human errors and unregulated data management are a substantial risk to the humanitarian workers but also to the communities they aim to protect via their interventions. In this work, we touch upon humanitarians' concerns with this new technology, and reflect on balancing those with the benefits of accuracy of interventions. We argue that the humanitarian sector must not overlook these technologies, as failing to adopt them, risks widening inequalities in access to data-driven decision-making and leaving vulnerable systems further behind. At the same time, it is essential to establish meaningful safeguards and promote local ownership to ensure that AI enhances resilience, rather than reinforcing existing power imbalances.

## Introduction

What are the implications of using artificial intelligence (AI) in the humanitarian sector? For those unfamiliar with digital technologies, AI is an impenetrable black box, dangerous to the humanitarian agencies' work. 1 For tech erudite, it is simply another tool to collect, aggregate, and distribute information, drawn from the Internet. As Radford explained, OpenAI trained its initial version of ChatGPT on a corpus of information available on social media, to output scenarios. 2 Given the recurrence of online data, the algorithm is now able to give us an infinite amount of information, summarize, analyze, and even make predictions; as long as we know what to ask. Integrating AI as a tool for information management can allow humanitarian organizations to evaluate its added value in conflict settings, and understand the trade-off of not being involved in this digital transformation.

This reflection is especially important to humanitarians because their interventions rely on computing large amount of data to increase their impact. Indeed, AI has already shown promising results in increasing the accuracy of health diagnostics in the field. 3 However, in a crisis context where security concerns are evolving to include data breaches, we will question humanitarians' position, with regard to sharing data online, and their responsibility regarding communities' safety.

## Learning Corpus

Humanitarian operations rely on accurate data to meet the specific needs of a targeted population. This understanding is crucial to coordinate between operating agencies and calibrate the scale of the needs. In emergency situations, first responders build their initial database on available online information, which in turn are refined by a set of assessments. In that context, online information from news outlets, social media, and others online platforms, becomes critical to navigate the initial steps of a humanitarian response, conducting analysis, describing risks, and testing scenarios. 4 In recovery and development contexts as well, the available set of data is also a determining element, to map population movements, track food distribution, provide medical diagnostics, and ensure quality reporting for further interventions.

Actors operating along the humanitarian-development nexus have started integrating digital tools to their activity to better manage emergencies. 5 The use of AI systems as a detection, prediction, and optimization tool, becomes a step up toward efficacy, and efficiency. Despite the controversies around digital humanitarianism described by Duffield, 6 local emergency agencies have used data mining to take back control over a crisis situation.

From a humanitarian perspective, the concerns with AI remain in the legitimacy of the data and the impact of inaccurate information could have in the field. As of today, digital tools, particularly the AI ones, are trained on the wrong contexts. Indeed, AI tools have a “World-Leading-Nations” confirmation bias because the majority of the available literature, and the overwhelming individual online presence, is coming from those geographies. More, it asserts ideas mimicking experts, distracting users from questioning the origin of an information. Calibrated by influx, and not legitimacy, AI shifted the definition of quality data.

## Scaling Up Impact

The capacity of digital technology to enhance the humanitarian response has already been proven. Thanks to the mass media coverage on the subject, the public has access to specific examples to how AI is scaling up humanitarian organizational efforts, which include: the Emergency Social Safety Net (ESSN) operated by International Federation of Red Cross and Red Crescent Societies in Türkiye, or even International Rescue Committee's usage of AI and machine learning to optimize its service delivery. 7 8 At the sectorial level as well, the humanitarian medical providers now have the possibility to use AI to improve their practice, analyzing within seconds X-rays using universal library of cases, or even precisely control a hospital environment to reduce the occurrence of some diseases. 9

This represents a support particularly critical for the medical sector, especially in crisis contexts like Syria, Ukraine, or Gaza, where the health personnel is often scarce and the resources running at a stretch. At employee's level as well, the impact of AI is potentially revolutionary. From writing and proofreading reports, generating maps and visuals, solving accounting issues, or even developing a new communication strategy. Early humanitarian staff has now the ability to optimize their work and improve the quality of their daily tasks. ChatGPT, Gemini, and Perplexity, among others, can professionally enhance anyone, with a simple command on a phone. With an unprecedented international Internet and mobile network coverage, smartphone ownerships have increased individuals' access to information and connection to the world like never before. 10 In conflict settings where the local work practices often differ from donors' “one-template-fits-all” standardized approach, AI technology becomes handy to easily and quickly meet the requirements. Saving time and money, humanitarians quickly find the added value to prompting part of their work.

However, this effortless gain in competences and individualization of the learning process might be where the security breach starts. Creating an isolated learning bubble, users cannot challenge the veracity of the learnt information, as often ill-equipped in front of a powerful algorithms. 10 Indeed, and contrary to agencies, which strengthen their digital security, individual staff are often misinformed on the dangers behind information breach and the scale deep fake can have, especially in vulnerable environments like a humanitarian crisis.

## Trade-Offs

Although phones are an important tool for humanitarians operating in a crisis environment, people's understanding of how they can be hacked and how their data can be sold, is still limited. Indeed, sensitive data can easily be sold by social media or AI platforms like ChatGPT. In that regard, the subject of AI ethics reached a critical point, as it touches upon the need to protect communities, and combat misinformation which can harm the veracity of a narrative. 11 These concerns are especially relevant in fragile contexts where the need for an accurate information is high, and fact-checking is a life-saving matter.

AI can improve humanitarians' interventions, but its centralized algorithm and unregulated access, are likely to decrease communities' safety in the process. In that case, can AI be used without inadvertently causing harm? In fragile environments, where institutions were toppled, who is securing field data, if not local individuals themselves. Knowing that ensuring data accuracy is a low priority for AI giants like OpenAI, 12 it is important to consider alternatives to secure individuals' data. In the absence of international regulatory framework, decentralizing AI to local data labs is important to consider, for individuals to take ownership of their narrative.

## Conclusion

In fragile settings, digital tools can connect the most secluded regions with a prevention campaign, alert those living in hard-to-reach areas on weather hazards, but it can also impair humanitarians' work with conflicting information, as they are often unable to confirm the legitimacy of the source of origin. Humanitarian organizations are often able to control remotely, AI-power solutions and increase their coverage. However, while everyone is focusing on strengthening regulations at the level of organizations, fewer are looking at preventive measures focusing on individuals' understanding of AI usage and risks. AI offers significant benefits, but if not correctly managed, it can compromise the very security and privacy of the individuals it aims to protect.
